# Chromatin-Remodeling Factor CHR5 Promotes Defense Gene Expression and SA Accumulation

**DOI:** 10.3390/plants15060967

**Published:** 2026-03-20

**Authors:** Xueru Liu, Ning Cui, Zhengxi Gong, Hainan Tian, Yuelin Zhang, Xin Li

**Affiliations:** 1 Michael Smith Laboratories, University of British Columbia, Vancouver, BC V6T 1Z4, Canada; xueru.liu@msl.ubc.ca (X.L.); cuining20061988@gmail.com (N.C.); zhengxig@student.ubc.ca (Z.G.); 2 Department of Botany, University of British Columbia, Vancouver, BC V6T 1Z4, Canada; 3 The College of Life Sciences, Sichuan University, Chengdu 610064, China; tianhainan2012@126.com

**Keywords:** plant immunity, CAMTA, CHR5, salicylic acid, NHP, chromatin-remodelling factor

## Abstract

Chromatin remodelers play essential roles in modulating nucleosome structure and enabling dynamic transcriptional control. Arabidopsis calmodulin-binding transcription activators CAMTA1/2/3 negatively regulate plant immunity by suppressing the expression of biosynthesis genes of major defence hormones salicylic acid (SA) and N-hydroxy-pipecolic acid (NHP). The autoimmunity of the *camta2/3* mutant is partially suppressed by loss of the NHP biosynthesis enzyme SAR deficient 4 (SARD4). During a forward genetic screen with the mildly autoimmune *camta2/3 sard4* mutant, we identified *chromatin-remodelling factor 5* (*chr5*) as its partial suppressor. The *chr5* single mutants displayed decreased SA biosynthesis and compromised basal immunity. Further RNA-sequencing with *chr5* defined immune-related genes that were downregulated in the mutants, including those involved in SA and NHP biosynthesis and signalling, PTI and ETI pathways. Our analysis highlights the roles of CHR5 in immune-specific chromatin remodelling events, contributing to transcriptional reprogramming during plant defence responses.

## 1. Introduction

Plants possess a sophisticated immune system to recognize pathogens and mount effective defence responses [1]. Recognition of pathogen-associated molecular patterns (PAMPs) by pattern-recognition receptors (PRRs) triggers the pattern-triggered immunity (PTI) [2]. To counteract, adapted pathogens secrete effectors that can modulate host immunity. In response, plants deploy resistance (R) proteins to recognize these effectors and activate strong effector-triggered immunity (ETI), typically associated with localized cell death [3,4]. PTI and ETI are interconnected and can mutually reinforce each other to ensure a robust local defence response [5,6,7,8]. These local defences can further trigger systemic acquired resistance (SAR) in uninfected systemic tissues, providing the plants with broad-spectrum and long-lasting protection.

Salicylic acid (SA) is an essential signalling hormone for the establishment of local defence and SAR [9,10]. The biosynthesis of SA in *Arabidopsis thaliana* is predominantly through the pathogen-induced isochorismate synthase (ICS) pathway [11]. ICS1 converts chorismate to isochorismate (IC), the transporter Enhanced Disease Susceptibility 5 (EDS5) exports IC from chloroplast to cytosol, and avrPphB Susceptible 3 (PBS3) conjugates glutamate to IC, which is finally converted to SA [12,13].

N-hydroxy-pipecolic acid (NHP) has been identified as an essential molecule for SAR [14,15]. Local defence induces NHP production, which acts as a mobile signal to establish SAR in distal tissue [9,14,15]. NHP is derived from L-lysine, which is processed to pipecolic acid (Pip) by AGD2-like defence response protein 1 (ALD1) and SAR-deficient 4 (SARD4) sequentially [16,17], followed by conversion from Pip to NHP by flavin-dependent monooxygenase 1 (FMO1) [14,15]. Of note, *ald1* and *fmo1* mutants show completely abolished NHP production and are unable to establish SAR responses, whereas *sard4* mutant displays partially reduced Pip and NHP levels, and mildly reduced SAR response [15,16,17]. This suggests that an unidentified SARD4-independent pathway contributes to Pip and NHP biosynthesis. NHP can prime transcriptional reprogramming in systemic tissue, largely overlapping with the SAR-responsive genes [15,18]. These genes include SA and NHP biosynthesis, receptors, receptor-like kinases, R proteins, mitogen-activated protein (MAP) kinases (MAPKs), and transcription factors, etc. In addition, SAR also associates with chromatin remodelling, which enhances chromatin accessibility at the defence gene regulatory regions [19].

Transcriptional regulation plays a central role in controlling immunity. Calmodulin-binding transcription activators (CAMTAs), CAMTA1, CAMTA2, and CAMTA3, are key transcription factors that regulate the expression of defence genes [20,21,22]. CAMTA3 (also named signal-responsive protein (SR1)) was identified as a negative regulator of immunity [23,24,25], which represses the SA biosynthesis. *camta3* knockout mutants display autoimmunity. CAMTA1 and CMATA2 are close homologs of CAMTA3. Triple mutant analysis revealed that they function additively to repress SA and NHP biosynthesis gene expression by regulating the master transcription factor SARD1 and calmodulin-binding protein 60-like g (CBP60g) [20,21]. The *camta1/2/3* triple mutant displays extremely autoimmune phenotypes, characterized by severe dwarfism, cell death, and enhanced resistance to pathogens [26]. The SA or NHP mutant, such as *ics1*, *fmo1*, partially compromises the *camta1/2/3* autoimmunity, while the *ics1 fmo1* double mutant nearly fully abolishes the autoimmune phenotypes [20,21].

Besides transcription factors, chromatin remodelers are also important for transcriptional control [27]. These ATP-dependent chromatin remodelers reposition, remove, or alter the composition of nucleosomes, which shape the accessibility of DNA to the transcriptional machinery and therefore modulate gene expression [28,29,30]. The SNF2 (Sucrose nonfermenting 2) family proteins are ATPase-domain containing chromatin remodelers conserved in eukaryotes. They use the energy from ATP hydrolysis to control the histone-DNA interactions and the accessibility of the genome [31,32]. Arabidopsis encodes 41 SNF2 chromatin remodelers, grouped into 18 subfamilies [33]. They have broad functions in growth, development, and stress responses [28].

The Chromodomain helicase DNA-Binding (CHD) proteins belong to one of the SNF2 subfamilies [34]. CHD1, one of the three subfamilies of CHD, contains only one protein in Arabidopsis, chromatin remodelling factor 5 (CHR5) [28,35]. CHR5 functions in both development and immunity [36,37,38]. CHR5 acts antagonistically with the CHD3 protein pickle (PKL) to regulate seed development gene expression, such as the *abscisic acid-insensitive 3* (*ABI3*) and *fusca3* (*FUS3*), by directly binding to their promoter regions. The *chr5* mutant shows decreased *ABI3* and *FUS3* expression and increased trimethylated lysine 27 of histone H3 (H3K27me3) modification on these genes during seed development [37]. In addition, the *chr5* mutant suppresses the autoimmunity of the gain-of-function R protein suppressor of *npr1-1*, constitutive 1 (*snc1*) [36]. CHR5 directly binds to the *SNC1* promoter region, positively regulates its expression, although how CHR5 affects *SNC1* expression remains unknown [38]. Furthermore, the *chr5* single mutant confers susceptibility to the virulent and avirulent pathogens, indicating CHR5 functions as a positive regulator for basal immunity, PTI, and ETI [36]. Micrococcal nuclease (MNase)—sequencing shows *chr5* mutant increases the genome-wide nucleosome occupancy on the gene promoters [36]. However, besides *SNC1*, the exact pathways and defence genes regulated by *CHR5* remain unclear.

In this study, we performed a forward genetic screen using the mild autoimmune mutant *camta2/3 sard4* and identified that loss-of-function of *CHR5* partially suppressed *camta2/3 sard4* autoimmunity. Further RNA-sequencing analysis on PAMP-induced Col-0 and *chr5* mutants revealed a broad range of genes involved in the SA and NHP biosynthesis and signalling pathways, PTI- and ETI-associated genes, were downregulated in *chr5* mutants. Collectively, our analysis revealed wide regulation of plant immune responses through CHR5-mediated chromatin remodelling.

## 2. Results

### 2.1. Identification of 296-1 Mutant as a Partial Suppressor of camta2/3 sard4

**Figure 1:**
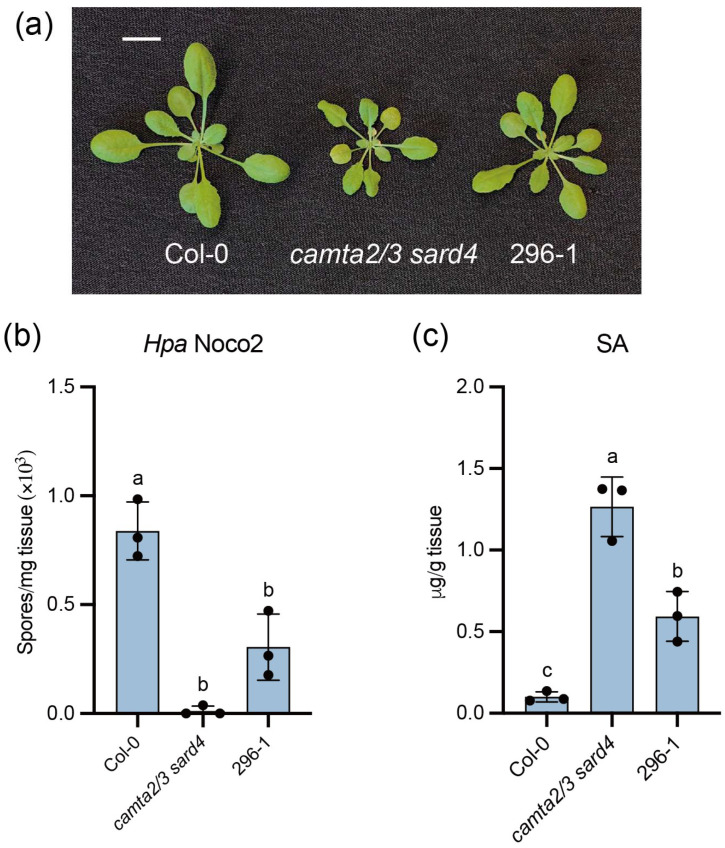
296-1 was identified as a partial suppressor of *camta2/3 sard4*. (**a**) The morphology of three-week-old plants of the indicated genotypes grown under long-day conditions. The scale bar is 1 cm. (**b**) The growth of the *Hpa* Noco2 conidiospores on the indicated genotypes. Three-week-old plants grown under long-day conditions were sprayed with 50,000 spores/mL *Hpa* Noco2 spores. The growth of the conidiospores was quantified seven days post-inoculation. This experiment was repeated twice with similar results. (**c**) Free SA levels of the indicated genotypes. The plant tissue from four-week-old plants grown under long-day conditions was harvested, and their SA levels were measured using HPLC. For (**b**,**c**), error bars represented the standard deviations (SD) of three biological replicates (*n* = 3). The letters indicated the statistical differences identified by one-way ANOVA with Tukey’s multiple comparisons test (*p* < 0.05). This experiment was repeated twice with similar results.

We previously conducted forward genetic screens on *camta1/2/3* autoimmune mutants to identify positive immune signalling components regulated by CAMTA transcription factors [21,39]. However, the strong autoimmunity and severe dwarf phenotype of *camta1/2/3* rendered partial suppressor phenotypes difficult to observe, especially for genes with functional redundancy or smaller contributions than the well-characterized single genes. To increase the screen sensitivity, we crossed *sard4* with *camta2/3* and generated *camta2/3 sard4* triple mutant, which maintains autoimmunity compared to wild-type (WT) Col-0 plants (Figure 1). To identify additional immune proteins regulated by CAMTA, we conducted an ethyl methane sulfonate (EMS) based forward genetic screen to look for suppressors of *camta2/3 sard4* autoimmunity. Here, we describe the partial suppressor mutant 296-1 identified.

Compared with *camta2/3 sard4*, the 296-1 plants displayed a larger size, but still slightly smaller than WT (Figure 1a). The 296-1 mutant supported more growth of the virulent oomycete pathogen *Hyaloperonospora abidopsidis* (*Hpa*) Noco2 compared to *camta2/3 sard4* (Figure 1b). Consistently, the accumulated SA in *camta2/3 sard4* was significantly reduced in 296-1 (Figure 1c). However, both *Hpa* Noco2 resistance and SA accumulation in 296-1 remained higher than WT (Figure 1b,c). Taken together, 296-1 partially suppressed the autoimmune phenotypes of *camta2/3 sard4*.

### 2.2. Mapping-by-Sequencing of 296-1

Mapping-by-sequencing was performed to identify the causal gene for the suppressor 296-1. When the 296-1 mutant was backcrossed to *camta2/3 sard4*, the F1 plants displayed *camta2/3 sard4*-like phenotypes, suggesting that 296-1 is recessive. In the F2 population, 123 plants resembled *camta2/3 sard4* and 34 plants resembled 296-1, supporting a single recessive gene mutation in 296-1 (3:1, χ^2^ = 0.936, *p* value = 0.33). Then, the plant tissue from the suppressor-like plants was bulk collected for whole-genome-sequencing (WGS). The single-nucleotide polymorphism (SNP) frequencies of the EMS-generated mutations were plotted to identify the linkage region.

**Figure 2:**
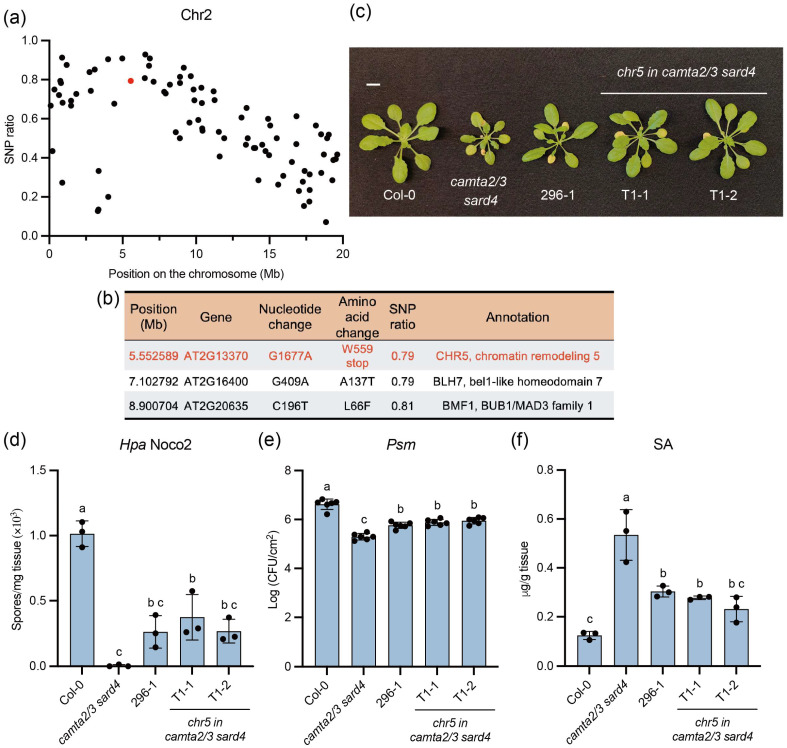
Mapping-by-sequencing of 296-1 and knocking out of *CHR5* suppressed the autoimmunity of *camta2/3 sard4*. (**a**) The SNP frequency on Chromosome 2 was calculated from the F2 mapping population of 296-1 mutants. The red dot indicated the SNP frequency of the *CHR5* mutation. (**b**) The list of the identified nonsynonymous mutations within the 3–9 Mb region on Chromosome 2. The *CHR5* mutation was indicated in red. (**c**) The morphology of four-week-old plants of the indicated genotypes grown under long-day conditions. The scale bar is 1 cm. (**d**) The growth of the *Hpa* Noco2 conidiospores on the indicated genotypes. Three-week-old plants grown under long-day conditions were sprayed with 50,000 spores/mL *Hpa* Noco2 spores. The growth of the conidiospores was quantified seven days post-inoculation. This experiment was repeated three times with similar results. (**e**) The growth of the *Psm* ES4326 on the indicated genotypes. The four-week-old plants grown under short-day conditions were infiltrated with *Psm* ES4326 (OD_600_ = 0.001), and the growth of the *Psm* ES4326 was quantified three days post-inoculation. Error bars represented the standard deviations (SD) of six replicates (*n* = 6). The letters indicated the statistical differences identified by one-way ANOVA with Tukey’s multiple comparisons test (*p* < 0.05). (**f**) Free SA levels of the indicated genotypes. The plant tissue from four-week-old plants grown under long-day conditions was harvested, and their SA levels were measured using HPLC. For (**d**,**f**), error bars represented the standard deviations (SD) of three replicates (*n* = 3). The letters indicated the statistical differences identified by one-way ANOVA with Tukey’s multiple comparisons test (*p* < 0.05). This experiment was repeated three times with similar results.

Among the five chromosomes, chromosome 2 showed a linkage region (Figure 2a and Figure S1). Due to the mild suppression phenotype of 296-1, some F2 plants collected for WGS may not be homozygous for the causal mutation, causing the SNP ratio peak in the linkage region not equaling 1. Then, the causal candidate mutations were filtered by selecting SNPs within the 3 Mb to 9 Mb region of chromosome 2 that had SNP frequencies larger than 0.7 and caused either nonsynonymous mutations on exons or mutations leading to splicing change. This analysis identified three candidate mutations (Figure 2b). Among them, a premature stop codon in Chromatin Remodelling Factor 5 (CHR5), caused by a G1677 to A mutation on exon 7 (Figure S2a), was prioritized for further study. CHR5 was previously reported as a positive regulator of plant immunity, and loss-of-function of *chr5* suppressed the autoimmunity of the gain-of-function NLR *Suppressor of npr1-1*, *Constitutive1* (*snc1-1*) [36].

### 2.3. Mutation in CHR5 Is the Causal Mutation for 296-1 Suppressor Mutant

To confirm whether the mutation on *CHR5* is causal for 296-1 mutant phenotypes, we knocked out *CHR5* in *camta2/3 sard4* using CRISPR/Cas9 (Figure S2a). Two homozygous deletion lines were obtained (Figure 2c and Figure S2b). Both *chr5* in *camta2/3 sard4* lines displayed similar plant sizes as the EMS mutant 296-1, larger than *camta2/3 sard4* (Figure 2c). When challenged with the virulent oomycete pathogen *Hpa* Noco2 and bacterial pathogen *Pseudomonas syringae* pv. *maculicola* (*Psm*) ES4326, both lines showed enhanced disease susceptibility compared to *camta2/3 sard4* (Figure 2d,e). The elevated SA levels were also reduced in these lines (Figure 2f). Similar to the partial suppressor 296-1, the disease resistance and SA accumulation in *chr5* in *camta2/3 sard4* were higher than Col-0 (Figure 2d–f).

**Figure 3:**
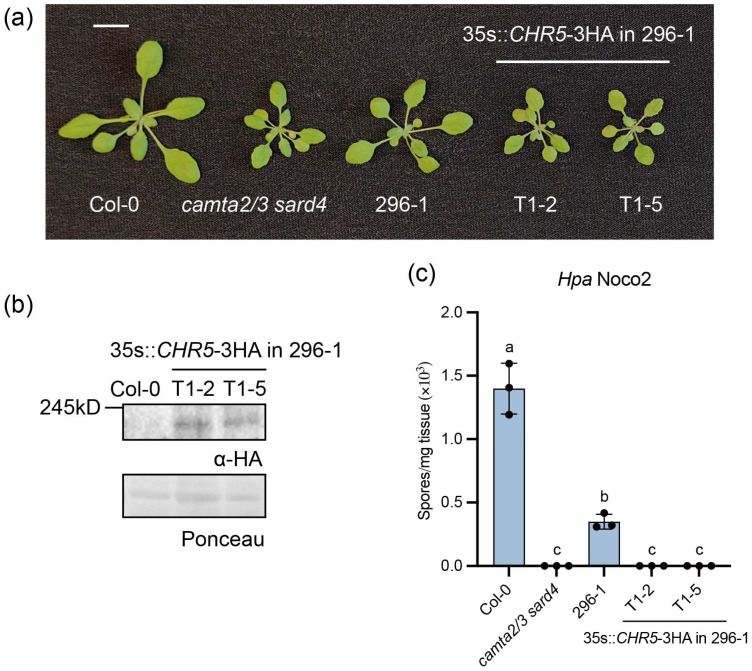
*CHR5-3HA* complemented the immunity defects of the 296-1 mutant. (**a**) The morphology of four-week-old plants of the indicated genotypes grown under long-day conditions. The scale bar is 1 cm. (**b**) Western blot showing the CHR5 protein in two independent lines of *35S::CHR5-3HA* transformed in 296-1. CHR5-3HA protein was probed with anti-HA antibody. Ponceau staining was used as the loading control. (**c**) The growth of the *Hpa* Noco2 conidiospores on the indicated genotypes. Three-week-old plants grown under long-day conditions were sprayed with 50,000 spores/mL *Hpa* Noco2 spores. The growth of the conidiospores was quantified seven days post-inoculation. Error bars represented the SD of three biological replicates (*n* = 3). The letters indicated the statistical differences identified by one-way ANOVA with Tukey’s multiple comparisons test (*p* < 0.05). This experiment was repeated three times with similar results.

To confirm the correct cloning of *CHR5*, a transgene complementation experiment was performed by transforming the 35S promoter-driven *CHR5* into the 296-1 mutant. Two independent lines were obtained (Figure 3a,b). In both lines, the transgenic plants became smaller as *camta2/3 sard4* (Figure 3a), and the enhanced *Hpa* Noco2 resistance was also restored (Figure 3c). Taken together, the early stop codon mutation in *CHR5* was the cause of the immune suppression phenotypes in 296-1 mutant. As six *chr5* mutant alleles were used before [36,37], we named the new early stop codon mutation in *chr5* in 296-1 as *chr5-7*.

### 2.4. The chr5 Single Mutants Display Compromised Basal Resistance

**Figure 4:**
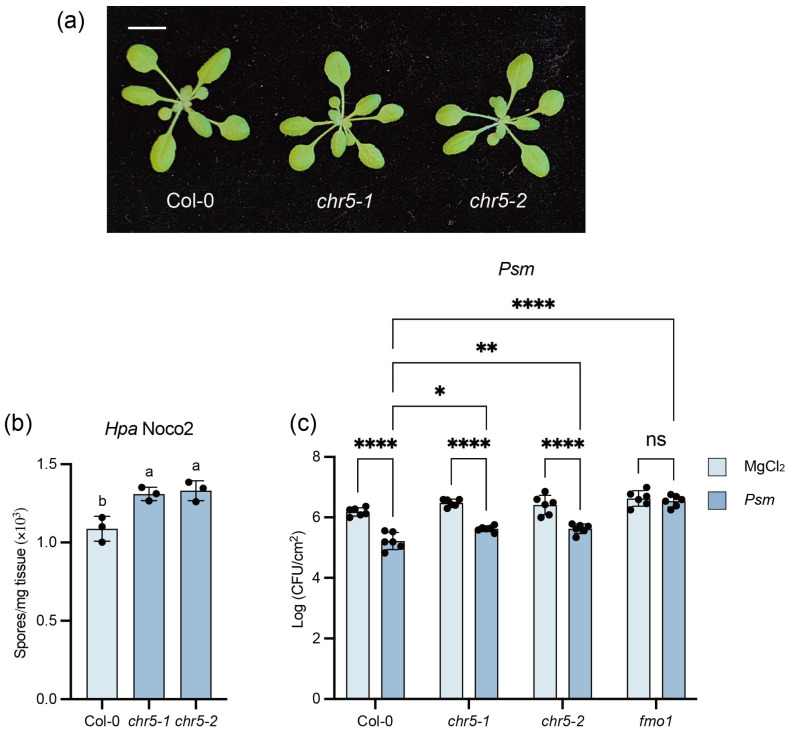
The *chr5* mutants showed compromised basal resistance. (**a**) The morphology of three-week-old plants of the indicated genotypes grown under long-day conditions. The scale bar is 1 cm. (**b**) The growth of the *Hpa* Noco2 conidiospores on the indicated genotypes. Three-week-old plants grown under long-day conditions were sprayed with 50,000 spores/mL *Hpa* Noco2 spores. The growth of the conidiospores was quantified seven days post-inoculation. Error bars represented the SD of three biological replicates (*n* = 3). The letters indicated the statistical differences identified by one-way ANOVA with Tukey’s multiple comparisons test (*p* < 0.05). This experiment was repeated three times with similar results. (**c**) The growth of the *Psm* ES4326 on the indicated genotypes upon treatment with MgCl_2_ or *Psm* ES4326. Two leaves from four-week-old plants grown under short-day conditions were infiltrated with 10 mM MgCl_2_ or *Psm* ES4326 (OD_600_ = 0.001). Two days after, the systemic leaves were infiltrated with *Psm* ES4326 (OD_600_ = 0.001), and the growth of the bacteria was quantified three days after. Error bars represented the SD of six replicates (*n* = 6). Statistically significant differences were identified by two-way ANOVA with Tukey’s multiple comparisons test (**** *p* < 0.0001, ** *p* < 0.01, * *p* < 0.05, ns: no significant difference). This experiment was repeated twice with similar results.

The previous study had examined the role of CHR5 in immune responses. Two T-DNA insertion lines, *chr5-1* (SALK_020296) and *chr5-2* (SAIL_504_D01), in the Col-0 background showed enhanced disease susceptibility to the virulent bacterial pathogens *Psm* ES4326, avirulent pathogens *Pseudomonos syringae* pv. *tomato* DC3000 *avrRpt2* and *Pst* DC3000 *avrRps4* as well as the type III secretion system deficient *Pst* DC3000 *hrcU*^-^. We further test these two *chr5* mutants (Figure 4a) with the virulent oomycete pathogen *Hpa* Noco2, which supported increased *Hpa* Noco2 growth compared to Col-0 (Figure 4b). These confirmed that CHR5 functions as a positive regulator in basal immunity, contributing to both PTI and ETI responses.

Furthermore, we examined the role of CHR5 in SAR. The plants were pretreated with *Psm* ES4326 on local leaves, followed by a second infection with *Psm* ES4326 on distal leaves. Compared to mock treatment, the *Psm* ES4326-pretreated Col-0 plants reduced *Psm* ES4326 growth in distal leaves, confirming the establishment of SAR. The SAR-deficient mutant *fmo1* was included as a positive control, which displayed no difference in the second *Psm* ES4326 growth with or without primary *Psm* ES4326 induction. Both *chr5-1* and *chr5-2* mutants still displayed the ability to establish SAR. However, the *Psm* ES4326 growth in distal leaves was higher than that of Col-0 following the *Psm* ES4326 induction (Figure 4c). This suggests that CHR5 contributes slightly to SAR.

### 2.5. Overexpression of CHR5 in Col-0 Leads to Enhanced Disease Resistance

**Figure 5:**
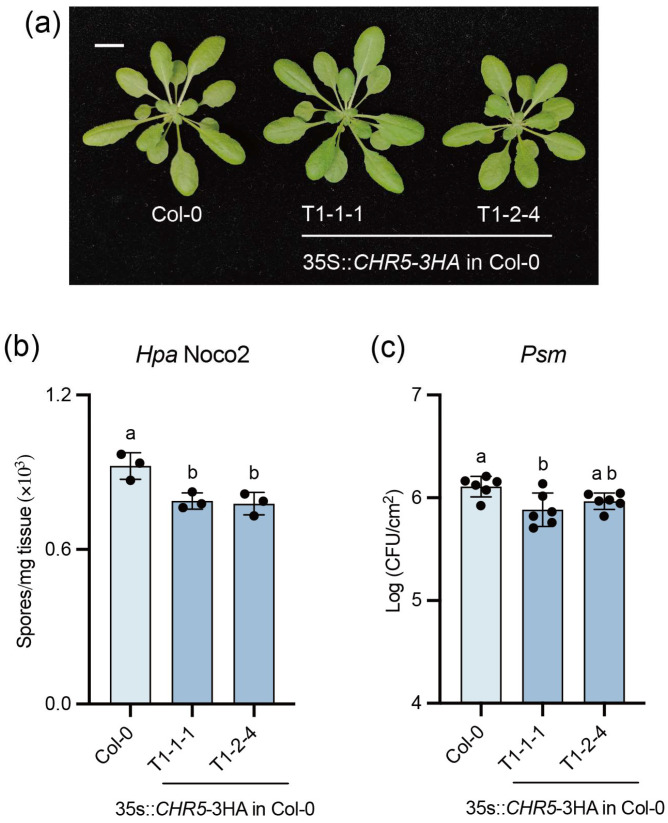
CHR5 overexpression leads to enhanced disease resistance. (**a**) The morphology of four-week-old plants of the indicated genotypes grown under long-day conditions. The scale bar is 1 cm. (**b**) The growth of the *Hpa* Noco2 conidiospores on the indicated genotypes. Three-week-old plants grown under long-day conditions were sprayed with 50,000 spores/mL *Hpa* Noco2 spores. The growth of the conidiospores was quantified seven days post-inoculation. Error bars represented the SD of three biological replicates (*n* = 3). The letters indicated the statistical differences identified by one-way ANOVA with Tukey’s multiple comparisons test (*p* < 0.05). This experiment was repeated twice with similar results. (**c**) The growth of the *Psm* ES4326 on the indicated genotypes. Two leaves from four-week-old plants grown under short-day conditions were infiltrated with *Psm* ES4326 (OD_600_ = 0.001). The growth of the bacteria was quantified three days after. Error bars represented the SD of six biological replicates (*n* = 6). The letters indicated the statistical differences identified by one-way ANOVA with Tukey’s multiple comparisons test (*p* < 0.05). This experiment was repeated twice with similar results.

We further generated the *CHR5* overexpression plants by transforming the 35S promoter-driven *CHR5* into Col-0. Two independent lines were obtained, which show similar morphology as WT (Figure 5a). When tested with *Hpa* Noco2 and *Psm* ES4326, both *CHR5* overexpression lines displayed lightly enhanced disease resistance (Figure 5b,c). This further supports that CHR5 functions as a positive regulator of plant defence responses.

### 2.6. CHR5 Regulates Defence Gene Expression

**Figure 6:**
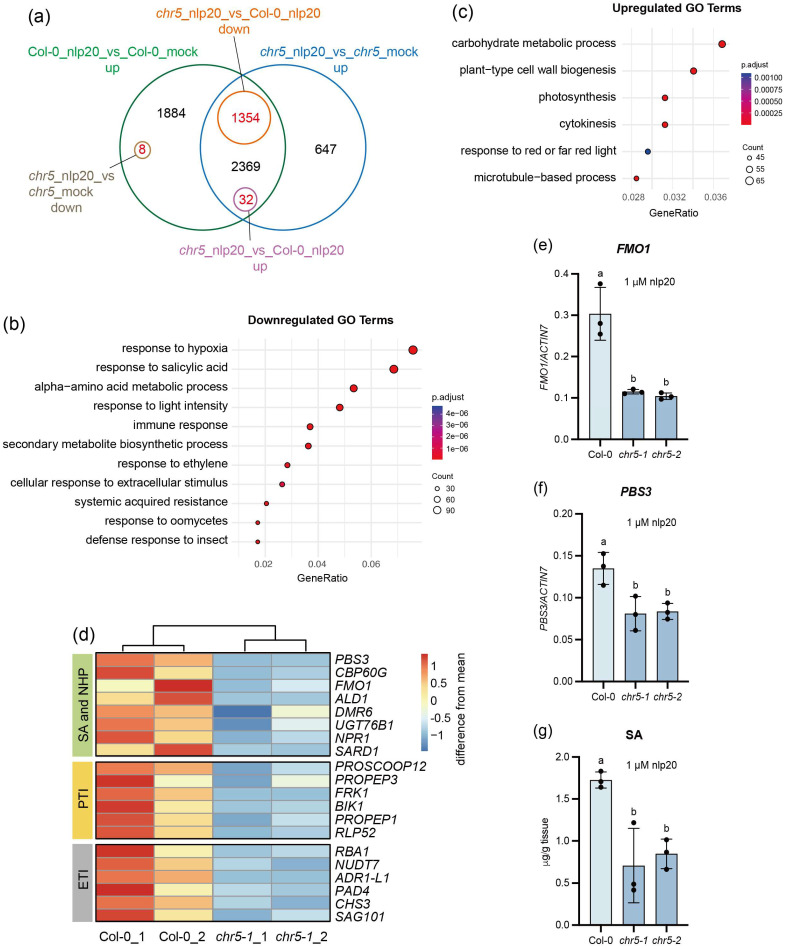
CHR5 positively regulates defence gene expression. (**a**) Venn diagram showing the number of DEGs in Col-0 and *chr5-1* by mock- or nlp20-treatment identified from the RNAseq dataset. (**b**) GO terms of biological process enriched among the downregulated DEGs in *chr5-1* mutant compared to Col-0 after treatment with nlp20. (**c**) GO terms of biological process enriched among the upregulated DEGs in *chr5-1* mutant compared to Col-0 after treatment with nlp20. (**d**) Heatmap of the defence genes downregulated in *chr5-1* mutant relative to Col-0 after treatment with nlp20. Each row represented a gene with the gene name shown on the right. Each column showed a biological replicate. (**e**,**f**) RT-qPCR result showed the expression levels of *FMO1* (**e**) and *PBS3* (**f**) in Col-0, *chr5-1*, and *chr5-2*. The 10-day-old plants grown on ½ MS plates were soaked with 1 μM nlp20 for 9 hours. The gene expression was normalized by *ACTIN7*. Error bars represented the SD of three biological replicates (*n* = 3). The letters indicated the statistical differences identified by one-way ANOVA with Tukey’s multiple comparisons test (*p* < 0.05). This experiment was repeated twice with similar results. (**g**) The free SA levels in Col-0, *chr5-1*, and *chr5-2* treated with 1 μM nlp20 for 24 hours. Four-week-old plants grown under short-day conditions were treated with 1 μM nlp20 for 24 hours and then harvested to measure the SA levels using HPLC. Error bars represented the SD of three biological replicates (*n* = 3). The letters indicated the statistical differences identified by one-way ANOVA with Tukey’s multiple comparisons test (*p* < 0.05). This experiment was repeated twice with similar results.

As CHR5 is a chromatin remodeler, we performed RNA sequencing (RNA-seq) analysis to explore its target genes. The Col-0 and *chr5-1* were treated with the PAMP ethylene-inducing peptide 1 (Nep1)-like protein 20 (nlp20) for 9 h to induce the defence gene expression. There are in total 5647 genes induced by nlp20 in Col-0 (|log_2_ fold change| ≥ 0.6, *p* ≤ 0.05) (Supplemental Table S2). Among them, 3755 genes were induced in both nlp20-treated Col-0 and nlp20-treated *chr5-1* mutant (Figure 6a). Compared to nlp20-treated Col-0, 1354 genes showed reduced induction in the *chr5-1* mutant, whereas 32 genes showed higher induction. In addition, 8 genes that were induced in nlp20-treated Col-0 were downregulated in nlp20-treated *chr5-1* (Figure 6a). Overall, 1394 (24.7%) nlp20-induced genes in Col-0 exhibited different expression in the *chr5-1* mutant.

A total of 3763 differentially expressed genes (DEGs) were identified between nlp20-induced Col-0 and nlp20-induced *chr5-1*, among which 1859 genes were upregulated, and 1904 genes were downregulated (|log_2_ fold change| ≥ 0.6, *p* ≤ 0.05) in *chr5-1* compared to Col-0 upon treatment with nlp20 (Supplemental Table S2). Gene ontology (GO) analysis of downregulated DEGs revealed enrichment of biological processes such as response to SA, immune response, response to molecules of bacterial origin, systemic acquired resistance, and response to oomycetes (Figure 6b), suggesting that CHR5 positively regulates defence gene expression. Moreover, the upregulated genes were primarily associated with processes such as carbohydrate metabolic process, cell wall biogenesis and photosynthesis, whereas defence-related processes were not enriched in the upregulated genes (Figure 6c).

More specifically, we identified many known immune genes regulated by CHR5 (Figure 6d). First, the expression of key genes involved in SA and NHP biosynthesis, including *PBS3*, *FMO1*, and *ALD1*, as well as the master transcription factors *SARD1* and *CBP60g* [40], was noticeably reduced in *chr5*. Secondly, some genes associated with PTI responses were repressed, including *PROSCOOP12* [41], *PROPEP1* [42], the receptor-like cytoplasmic kinase (RLCK) *Botrytis-Induced Kinase 1* (*BIK1*) [43], the RLK *Flg22-Induced Receptor Kinase 1* (*FRK1*), as well as *Receptor-Like Protein* (*RLP52*) [44]. In addition, components of ETI signalling, including *Phytoalexin Deficient 4* (*PAD4*), *Senescence-Associated Gene 101* (*SAG101*), helper NLR *Activated Disease Resistance 1* (*ADR1-L1*) [45], R protein *Response to the bacterial type III effector protein HopBA1* (*RBA1*) [46] and *Chilling Sensitive 3* (*CHS3*) [47], were also downregulated in *chr5*. These highlight the broad impact of CHR5 on transcriptional regulation of defence genes in plant immunity.

Furthermore, the reverse transcription quantitative PCR (RT-qPCR) was performed to confirm differential gene expression in the *chr5* mutant. Indeed, the expression of NHP biosynthesis gene *FMO1* (Figure 6e) and SA biosynthesis gene *PBS3* (Figure 6f) was decreased in both knockout lines of *chr5* compared to Col-0 after treatment with nlp20, validating the RNA-seq results.

Lastly, to determine whether the decreased gene expression affects the SA biosynthesis, the nlp20-induced SA accumulation was measured. As shown in Figure 6g, the SA accumulation was significantly lower in *chr5-1* and *chr5-2* relative to Col-0 after nlp20 treatment. This reduced SA likely accounts for the compromised basal resistance and mild SAR defects observed in *chr5* mutants (Figure 4).

## 3. Discussion

Previous studies have shown that the autoimmunity of *bon1* (*bonzai1*) and *snc1* depends on CHR5 [36], which regulates *SNC1* gene expression [38]. In this study, *chr5* mutants also suppressed the autoimmunity of *camta2/3 sard4* (Figure 2), and *chr5* single mutants displayed compromised basal resistance (Figure 4), which indicates CHR5 plays a broad role in regulating immune response. 

The RNAseq analysis was performed on Col-0 and *chr5-1* to identify the genes regulated by CHR5. We used nlp20 instead of flg22 to induce defence gene expression, as nlp20 induces a stronger transcriptional response in Col-0 under our experimental conditions [7]. To evaluate the induction of transcriptional change by nlp20 in our study, we compared our nlp20-treated Col-0 RNAseq dataset with the published microarray dataset, in which 5-week-old Col-0 plants were infiltrated with 1 μM NLP_Pp_ protein from the *Phytophthora parasitica* for 4 hours [48]. A substantial overlap between the two datasets was observed. NLP_Pp_ induced 531 genes (|log_2_ fold change| ≥ 1, *p* < 0.05) at 4 h. Among these genes, 462 genes (86.6%) (|log_2_ fold change| ≥ 1, *p* < 0.05) were also upregulated in our nlp20-treated Col-0 compared to mock treatment and only 3 genes were downregulated (Table S4). Interestingly, among the 462 commonly upregulated genes, 66 genes (14.3%) showed reduced induction in *chr5-1* mutant compared to Col-0 (Table S4). Together, this overlap supports the reliability of our RNAseq data and confirms that nlp20 treatment in our experiment triggers transcriptional changes consistent with the previously reported study.

From our transcriptome analysis, nlp20-induced *SNC1* expression was indeed reduced in *chr5* mutants (Figure S3). Besides *SNC1*, we also identified more immune genes regulated by CHR5, including those involved in SA and NHP biosynthesis and signalling, PTI and ETI pathways (Figure 6). It should be noted that the fold changes of these immune genes in *chr5* mutants relative to Col-0 were modest, mostly less than 2.5-fold (Supplemental Table S2). This mild reduction likely accounts for the impaired basal resistance but very mild SAR defects in *chr5* mutants (Figure 4), suggesting that the small reduction in these immune genes expression is insufficient to abolish SAR, or it is too subtle to be detected using our infection methods.

Notably, a recent study analyzed the genome-wide chromatin accessibility during defence responses using transposase-accessible chromatin followed by sequencing (ATAC-seq), which revealed that promoter regions of *SARD1*, *ICS1*, *EDS5*, *PBS3*, *ALD1*, *SARD4*, and *FMO1* all showed increased chromatin accessibility upon pathogen infection [49]. It will be interesting to determine whether CHR5 directly regulates the chromatin accessibility of these loci during immune activation. In addition, whether CHR5 acts with the transcriptional machinery to control immune gene expression remains to be elucidated.

Chromatin remodelers also coordinate with histone modifications to fine-tune the nucleosome positioning and transcriptional control [28]. For example, the plant H3K27 demethylase Relative of Early Flowering 6 (REF6) recognizes a specific genomic motif and recruits the chromatin remodeler BRAHMA (BRM). They co-regulate many genes’ expression across the genome [50]. An independent study showed that the histone deacetylase (HDAC) HD2C interacts with BRM and cooperatively controls heat-responsive gene expression [51]. Another chromatin remodeler, PKL, directly acts on the genes enriched with H3K27me3 modification [52]. The rice CHD3 protein CHR729 binds to and also regulates the H3K4me2 and H3K27me3 on the target genes during plant development [53]. In *chr5* mutants, the H3K27me3 levels on *FUS3* and *ABI3* were increased during seed development [37]. Although CHR5 was enriched in the promoter region of *SNC1*, neither the genome accessibility nor the H3K4me3 modification at *SNC1* was changed in *chr5* mutants [38]. How CHR5 regulates *SNC1* transcripts remains unclear. Moreover, whether CHR5 directly binds to the differentially expressed genes identified from our RNA-seq data and whether CHR5 affects histone modifications at these loci remains to be determined. Future study by chromatin immunoprecipitation (ChIP) sequencing could help identify these exact regulatory details. Also, whether CHR5 coordinates with any histone modification enzymes to co-regulate gene expression needs further investigation.

The activity of chromatin remodelers could be regulated by post-translational modifications. For instance, BRM represses the transcription factor *ABA Insensitive 5* (*ABI5*) expression in the absence of abscisic acid (ABA) [54]. Increased ABA activates Sucrose non-fermenting 1-Related protein Kinases 2 (SnRK2s), which directly phosphorylate BRM, inhibiting its activity and thereby derepressing *ABI5* expression. Conversely, under low ABA conditions, the protein phosphatases type 2C (PP2Cs) dephosphorylate BRM to restore its repressive function [55]. Although CHR5 functions in seed development and stress response, how CHR5 activity is controlled remains unclear. Interestingly, one recent study analyzed the chromatin phosphoproteome in Col-0, *mitogen-activated protein kinase* (*mpk3*), *mpk4*, and *mpk6* mutants with or without flg22 treatment and identified potential chromatin-associated targets of MAPKs during defence response [56]. A CHR5-derived phosphopeptide, ‘KTEYFVPS(t)*PLLEGTSAQVR’, showed phosphorylation dependent on both flg22 and MPK3/MPK4/MPK6, which indicates this ‘T*P’ motif may be important for CHR5 activity in PTI response. In addition, CHR5 contains 21 predicted MAPK docking sites [56], suggesting that additional MAPKs may contribute to its phosphorylation. Notably, flg22 treatment decreased the phosphorylation of ‘KTEYFVPS(t)*PLLEGTSAQVR’ in WT, indicating phosphatases likely dephosphorylate CHR5 as well. Further studies will be required to dissect which kinases and phosphatases target CHR5 and how phosphorylation modulates CHR5 activity in gene transcriptional control.

## 4. Experimental Procedures

### 4.1. Plant Materials and Growth Conditions

The *Arabidopsis* plants in this study are in the Col-0 ecotype background. The *camta2/3* [26], *sard4-5* (GABI_428E01) [16], *chr5-1* (SALK_020296), *chr5-2* (SAIL_504_D01) [36,37] were described previously. *camta2/3* and *sard4-5* were crossed to generate *camta2/3 sard4*.

The two independent lines of *chr5* in *camta2/3 sard4* T1-1 and T1-2 were generated by transforming the CRISPR-Cas9 system [57], *pBEE* vector containing two gRNAs targeting *CHR5* into *camta2/3 sard4*. The T1 plants were selected from the soil by spraying Basta. The primers used for genotyping the plants with deletions in *chr5* were listed in Supplemental Table S1. The *CHR5*-edited plants were confirmed in T2 to obtain homozygous mutants.

The two independent lines of *35S::CHR5-3HA* in 296-1 were generated by transforming the *pCambia1300-CHR5-3HA* into the 296-1 mutants. The T1 plants were selected on ½ MS plates (PhytoTech Labs, Lenexa, KS, USA) containing hygromycin (MilliporeSigma, Oakville, ON, Canada).

For plants grown on soil under long-day conditions, the seeds were sterilized with bleach, and the germinated plants were grown with 16 h light and 8 h dark under 22 °C room temperature. For seedlings on half-strength Murashige and Skoog (½ MS) plates, the seeds were sterilized with bleach and planted on plates under long-day conditions. For plants grown on soil under short-day conditions, the seeds were germinated under long-day conditions, and then transferred to grow with 12 h light and 12 h dark under 22 °C room temperature.

### 4.2. Construction of Plasmids

To generate the *CHR5* CRISPR-Cas9 construct, the genomic sequence of *CHR5* was subjected to MMEJ-KO http://skl.scau.edu.cn/mmejko/ (accessed on 9 April 2024) to identify two gRNAs targeting *CHR5*. The primer pair CHR5-BsFF0 and CHR5-BsRR0 and the *pCBC-DT1DT2* template were used to amplify the fragment containing two gRNAs, and then inserted into the *pBEE* construct by the BsaI enzyme.

To generate the *pCambia1300-CHR5-3HA* construct, the CDS sequence of *CHR5* was amplified using primer pair CHR5-KpnI-35sF and CHR5-SpeI-nsR. The fragment was then inserted into the *pCambia1300-3HA* construct.

The primers used were listed in Supplemental Table S1.

### 4.3. EMS Mutagenesis and Mapping-by-Sequencing

Ethyl methanesulfonate (EMS) mutagenesis was performed as described [58]. Briefly, around 5000 *camta2/3 sard4* seeds were mutagenized by EMS and plated on ½ MS plates. The seeds from 16 M1 plants were bulk harvested as a population. From each pool, roughly 400 M2 progenies were screened to look for potential suppressors of *camta2/3 sard4* with increased plant size. The selected mutants were advanced to M3 for confirmation of the phenotype.

Mapping-by-sequencing was performed as previously described [39]. The 296-1 mutant was backcrossed with *camta2/3 sard4*. F1 plants showed *camta2/3 sard4*-like size. In F2, plant tissue from 34 plants showing a similar size as 296-1 was bulk harvested for whole-genome-sequencing by Illumina (Novogene, Beijing, China). After sequencing, the raw reads were processed by the pipeline built on the Genome Analysis Toolkit (GATK v.3.5-0). After identifying the single-nucleotide polymorphisms (SNPs), their allele frequency distributions were analyzed on each chromosome to find the linkage region.

### 4.4. Pathogen Infection

Three-week-old plants grown under long-day conditions were sprayed with *Hyaloperonospora arabidopsidis* (*Hpa*) Noco2 spore suspension (50,000 spores/mL). The plants were covered with transparent lids to keep high humidity and grown at 18 °C under short-day conditions for seven days. Then the spores from above-ground tissue were collected in H_2_O and counted using the hemocytometer. Each plant was used as a biological repeat. For each experiment, three biological repeats were used for each genotype, and eight technical repeats were performed for each biological repeat. Each experiment was repeated three times with similar results.

Four-week-old plants grown under short-day conditions were used for *Pseudomonas syringae pv. maculicola* (*Psm*) ES4326 infection. Two leaves from each plant were infiltrated with *Psm* ES4326 in 10 mM MgCl_2_ at OD_600_ = 0.001. The leaf discs were collected at Day 3 post-infiltration to assess the bacterial growth. Each plant was used as a biological repeat. The leaf discs were ground in 10 mM MgCl_2_ and serially diluted. Dilutions were then plated on LB plates with streptomycin and grown at 28 °C for one day. The colony-forming units were then counted.

Four-week-old plants grown under short-day conditions were used for the SAR assay. Two local leaves from each plant were infiltrated with 10 mM MgCl_2_ as a mock control or *Psm* ES4326 in 10 mM MgCl_2_ at OD_600_ = 0.001. Two days after, two systemic leaves were infiltrated with *Psm* ES4326 in 10 mM MgCl_2_ at OD_600_ = 0.001. The leaf discs from the infiltrated systemic leaves were collected at Day 3 post-infiltration to assess the bacterial growth as described above.

For each *Psm* ES4326 infection experiment, six biological repeats were used for each genotype, and one technical repeat was performed for each biological repeat. Each experiment was repeated twice or three times with similar results.

### 4.5. SA Extraction and Measurement by HPLC

SA was extracted and quantified as described [59]. Three leaves from four-week-old plants grown under short-day conditions were infiltrated with 1 μM nlp20. Leaves from each plant were harvested 24 h after infiltration for SA quantification using HPLC. For each experiment, three biological repeats were used for each genotype, and one technical repeat was performed for each biological repeat. Each experiment was repeated twice or three times with similar results.

### 4.6. RNA Sequencing and Data Analysis

The 10-day-old seedlings grown on ½ MS plates under long-day conditions were carefully pulled out from the plates and placed in H_2_O in 6-well plates to recover overnight. The next day, H_2_O was removed and replaced with 1 μM nlp20. After 9 h, the plant tissue was collected and frozen immediately in liquid nitrogen.

RNA-seq library was generated, and quality assessed by BGI-T7 (Personalbio, Shanghai, China), which produces roughly 20–45 million paired-end clean reads for each plant sample. The resulting sequences were mapped to the *Arabidopsis thaliana* reference genome (TAIR 11) with HISAT2 v2.2.0 under default settings [60]. The alignment files were processed using SAMtools v1.10 [61], and gene counts were obtained with featureCounts [62]. Differentially expressed genes (DEGs) were identified with DESeq2 v1.28.1 [63] with the criteria |log_2_(fold change)| ≥ 0.6, adjusted *p* value (padj) ≤ 0.05. Gene Ontology (GO) term enrichment was evaluated by Fisher’s exact test with Blast2GO annotations [64]. Gene expression patterns were visualized using R packages, including ComplexHeatmap (v2.16.0) [65], UpSetR (v1.4.0) [66], and ggplot2 (v3.5.1) [67].

### 4.7. Quantitative RT-PCR

The plant growth and treatment were similar as described above for RNA-seq. Total RNA was extracted using the EZ-10 Spin Column Plant RNA Miniprep Kit (BioBasic, Markham, ON, Canada). Around 1 μg of total RNA for each sample was reverse transcribed using the OneScript^®^ Reverse Transcriptase (ABM, Richmond, BC, Canada). qPCR was performed using SYBR Premix Ex Taq II (Takara, Kyoto, Japan). The primers used were listed in Supplemental Table S1. For each experiment, three biological repeats were used for each genotype, and two technical repeats were performed for each biological repeat. Each experiment was repeated twice with similar results.

## Data Availability

All data in this study are presented in the manuscript and Supplemental Materials.

## Supplementary Materials

The following supporting information can be downloaded at: https://www.mdpi.com/article/10.3390/plants15060967/s1, Figure S1: Mapping-by-sequencing of 296-1. (a–d) The SNP frequency on Chromosome 1, 3, 4, 5 calculated from the F2 mapping population of 296-1 mutant; Figure S2: Knocking out of *CHR5* in *camta2/3 sard4* by CRISPR/Cas9. (a) The gene structure of *CHR5.* Black boxes indicate the exons. Lines indicate the introns. The red arrows point out the two gRNAs used for CRISPR knock-out. Blue arrows show the primers used for genotyping the deletions on *CHR5*. The brown arrow points out the mutation of *CHR5* identified in the 296-1 mutants. The scale is 500 bp. (b) The genotyping results of two independent knock-out lines of *CHR5* mutants in *camta2/3 sard4*; Figure S3: *SNC1* expression in Col-0 and *chr5-1* mutant. *SNC1* transcript levels from the RNA-seq data in Col-0 and *chr5-1* with or without 1 μM nlp20 treatment. Error bars represented the SD of two replicates (*n* = 2). The letters indicated the statistical differences identified by two-way ANOVA with Tukey’s multiple comparisons test (*** *p* < 0.001, ** *p* < 0.01, * *p* < 0.05, ‘ns’ indicates no significant difference); Table S1: Primers used in this study; Table S2: Differentially expressed genes identified for the following comparisons: mock vs. 1 μM nlp20-treated Col-0, mock or 1 μM nlp20-treated *chr5-1* mutants, and 1 μM nlp20-treated Col-0 and 1 μM nlp20-treated *chr5-1* mutants using the criteria |log_2_(fold change)| ≥ 0.6, adjusted *p* value (padj) ≤ 0.05; Table S3: Differentially expressed genes identified for mock-treated Col-0 vs. mock-treated *chr5-1* using the criteria |log_2_(fold change)| ≥ 0.6, adjusted *p* value (padj) ≤ 0.05; Table S4: Comparison of 1 μM nlp20-induced genes at 9 h in Col-0 from this study and the 1 μM NLP_Pp_-induced genes at 4 h in Col-0 from a published microarray dataset.
