# Supplementary material for: Chromatin-Remodeling Factor CHR5 Promotes Defense Gene Expression and SA Accumulation
Source: Plants (Basel). 2026 Mar 20;15(6):967. doi: 10.3390/plants15060967 (PMC13030818; doi:10.3390/plants15060967)
Supplement: Supplementary file 1 [file plants-15-00967-s001.zip › plants-4086500-supp-figs.pdf]

Supplementary Materials

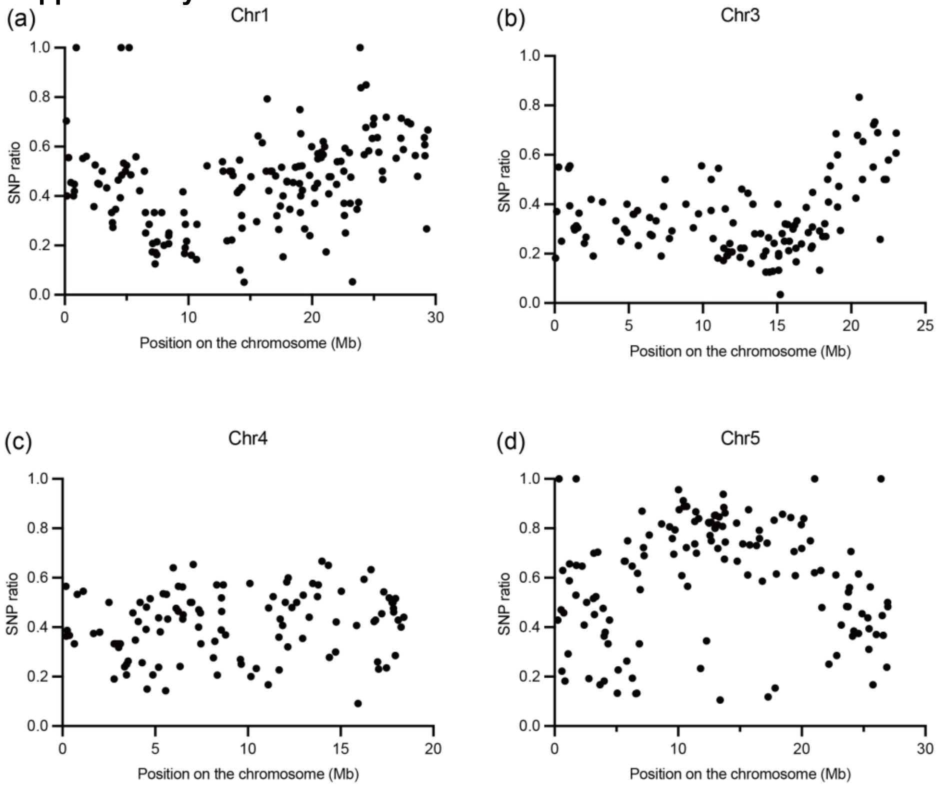

Figure S1. Mapping-by-sequencing of 296-1.

(a-d) The SNP frequency on Chromosome 1, 3, 4, 5 calculated from the F2 mapping population of 296-1 mutant.

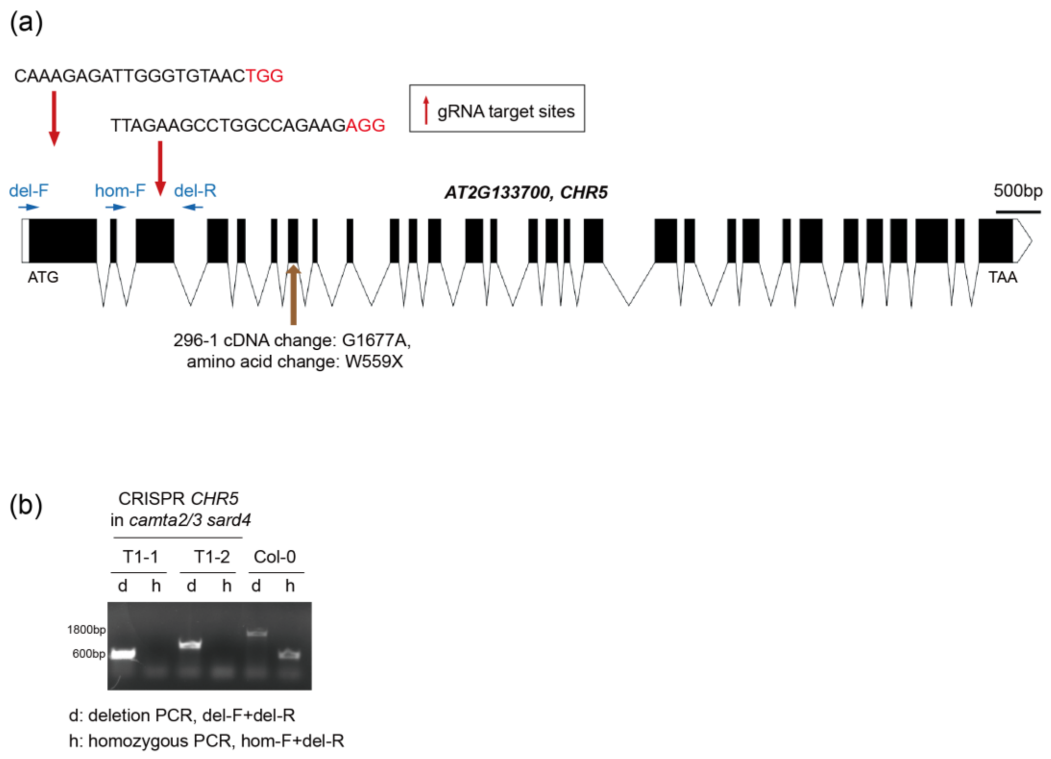

Figure S2. Knocking out of *CHR5* in *camta2/3 sard4* by CRISPR/Cas9.

(a) The gene structure of *CHR5*. Black boxes indicate the exons. Lines indicate the introns. The red arrows point out the two gRNAs used for CRISPR knock-out. Blue arrows show the primers used for genotyping the deletions on *CHR5*. The brown arrow points out the mutation of *CHR5* identified in the 296-1 mutants. The scale is 500bp.

(b) The genotyping results of two independent knock-out lines of *CHR5* mutants in *camta2/3 sard4*.

(a)

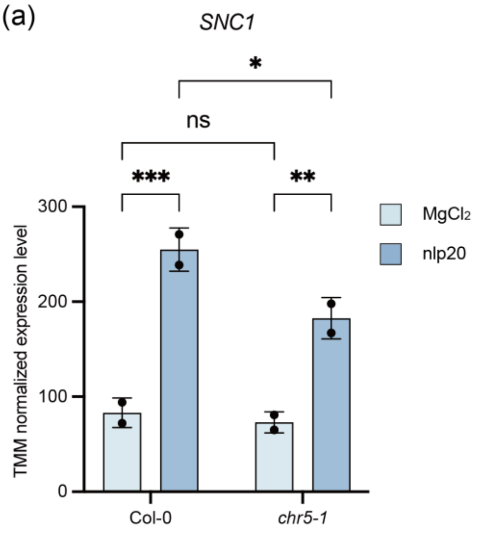

Figure S3. *SNC1* expression in Col-0 and *chr5-1* mutant.

(a) *SNC1* transcripts levels from the RNA-seq data in Col-0 and *chr5-1* with or without 1µM nlp20 treatment. Error bars represented the SD of three replicates (n = 2). The letters indicated the statistical differences identified by two-way ANOVA with Tukey's multiple comparisons test (***P < 0.001, **P < 0.01, *P < 0.05, 'ns' indicates no significant difference).
